# Platelet-derived growth factor receptor α in hepatocellular carcinoma is a prognostic marker independent of underlying liver cirrhosis

**DOI:** 10.18632/oncotarget.17134

**Published:** 2017-04-17

**Authors:** Jung-Hwan Yu, Joon Mee Kim, Ja Kyung Kim, Suk Jin Choi, Kwan Sik Lee, Jin-Woo Lee, Hye Young Chang, Jung Il Lee

**Affiliations:** ^1^ Department of Internal Medicine, Yonsei University College of Medicine, Seoul, Republic of Korea; ^2^ Department of Pathology, Inha University School of Medicine, Incheon, Republic of Korea; ^3^ Department of Internal Medicine, Inha University School of Medicine, Incheon, Republic of Korea; ^4^ Medical Research Center, Gangnam Severance Hospital, Yonsei University College of Medicine, Seoul, Republic of Korea

**Keywords:** platelet-derived growth factor receptor α, liver fibrosis, liver cirrhosis, hepatic stellate cell, hepatocellular carcinoma

## Abstract

**Background and Aims:**

Platelet-derived growth factor receptor alpha (PDGFRα) is suggested as a prognosis marker for hepatocellular carcinoma (HCC). Since PDGFRα is also known as a marker for activated hepatic stellate cells (HSCs), this study aimed to investigate whether PDGFRα expression in HCC was dependent on the background liver fibrous condition.

**Results:**

Strong PDGFRα expression in the tumor lesions was associated with decreased survival after curative HCC resection. Expression of PDGFRα in the tumor correlated with increased *collagen α1(I)*, *lecithin retinol acyltransferase*, and *smooth muscle α-actin* suggesting increased HSCs in tumor sites. The expression of PDGFRα in the tumor sites was associated neither with underlying liver fibrosis/cirrhosis nor with the expression of PDGFRα in adjacent non-tumor sites of the liver.

**Materials and Methods:**

Patients with HCC who underwent liver resection as curative treatment were included in this study. Using liver samples of 95 patients, tissue microarray was constructed and immunohistochemical study of PDGFRα was conducted in both tumor and non-tumor sites. PDGFRα expression in tumor and matching non-tumor sites was compared. Freshly frozen liver tissue specimens of 16 HCC patients were used for gene expression analysis of PDGFRα and fibrosis related genes.

**Conclusions:**

Our results suggest that PDGFRα overexpression in HCC is a prognostic marker independent of adjacent non-tumor site liver fibrosis status.

## INTRODUCTION

Chronic liver injury due to infectious, inflammatory or metabolic disorders often results in liver fibrosis and cirrhosis, which may predispose to hepatocellular carcinoma (HCC) [1, 2]. Although liver fibrosis and cirrhosis are well known risk factors for HCC, contribution of fibrous microenvironment to liver carcinogenesis has not been clearly elucidated. A molecular profiling study of HCC and adjacent non-tumor site reported that instead of the tumor itself, the gene signature of the adjacent non-tumor site contained important molecular information on HCC recurrence and prognosis [3]. This supports the possible significant role of stromal microenvironment to HCC carcinogenesis or progression.

Platelet derived growth factors (PDGFs) are potent mitogen for hepatic stellate cells (HSCs) which comprises an important cellular component in liver fibrosis and cirrhosis [4, 5]. The PDGF ligand family, PDGF-A, B, C, and D transmit extracellular signals through tyrosine kinase receptors which consist of two subunits, platelet-derived growth factor receptor (PDGFR) α and PDGFRβ [6, 7]. Increased PDGFRα is noticeable in cirrhotic liver and its primary expression is reported to be in HSCs [8]. Stimulation of PDGFR and overexpression of PDGF ligands may induce HSC proliferation [9–12]. Interestingly, overexpression of PDGF-C in hepatocytes and stimulation of PDGFRα results in not only liver fibrosis but eventually development of HCC, suggesting the significant role of PDGFRα in liver fibrosis and HCC tumorigenesis [13]. However, cellular target of PDGF-C, on which PDGFRα should be abundant, is still not clear. In non-cancerous condition, expression of PDGFRα is noticed on non-parenchymal liver cells, mainly HSCs. Therefore it can be hypothesized that overexpressed PDGF-C would stimulate PDGFRα on HSCs, which in turn may lead to the activation and proliferation of the cells. These activated HSCs might contribute PDGFRα over-expression in HCC tumor sites. However, link between activation, proliferation of HSCs and HCC formation is still not clear.

Cellular source of PDGFRα is also under dispute. Increase of PDGFRα expression has been reported in cancerous hepatocytes [14], while another study demonstrated that they were non-parenchymal cells in tumor sites where PDGFRα were expressed [15]. In addition, although HCC is often preceded by liver fibrosis where increased PDGFRα expression is frequently detected, association of PDGFRα up-regulation in HCC and the condition of adjacent non-tumor site is to be more thoroughly investigated. If PDGFRα expression were to be associated with the status of underlying liver disease, it could have been suggested that characteristics of HCC were to be dependent on the background liver condition.

This study aimed to investigate whether PDGFRα expression in HCC is associated with the status of the underlying liver disease by assessing PDGFRα expression on tumor and matching non-tumor sites.

## RESULTS

### Patient characteristics

Demographic findings of patients that were included in immunohistochemistry (IHC) analysis of PDGFRα are described in Table 1. Patients were 29-75 years in age (range, 53.2 ± 10.0), and mean follow-up time after surgery was 50.0 ± 39.3 months (range, 0-108). The etiology of underlying liver disease was HBV in 73 (76.8%), HCV in 6 (6.3%), alcohol in 3 (3.2%) and others in 13 (13.7%) patients. Pathologic liver cirrhosis, determined from histologic evaluation of liver non-tumor site, was identified in 64 (67.4%) patients.

**Table 1:** Baseline characteristics of patients that underwent liver resection due to HCC

Variables	n=95
**Age, years, median (range)**	54 (29-75)
**Gender (M:F)**	78:17
**Etiology of Liver Disease**	
HBV	73 (76.8)
HCV	6 (6.3)
Alcohol	3 (3.2)
Others	13 (13.7)
**Serum AFP, ng/mL (%)**	
≤200	58 (61.1)
>200	37 (38.9)
**Tumor number (%)**	
Single	13 (13.7)
Multiple	82 (86.3)
**Tumor size**	
≤5cm	65 (68.4)
>5cm	30 (31.6)
**Existence of satellite nodule**	10 (10.5)
**Existence of microvascular invasion (%)**	56 (55.4)
**Existence of macrovascular invasion (%)**	2 (2.1)
**Edmonson Grade (%)**	
Grade 1, 2	46 (48.4)
Grade 3, 4	49 (51.6)
**Existence of Pathologic Cirrhosis (%)**	**64 (67.4)**

AFP, alpha-fetoprotein.

Clinicopathologic characteristics of the patients were compared according to PDGFRα expression patterns (Table 2). Patients with strong PDGFRα expression on HCC showed similar gender ratio, higher proportion of patients with AFP >200 ng/mL, and lower portion of patients with liver cirrhosis compared with patients with either no or moderate PDGRα stain.

**Table 2:** Association between baseline clinicopathologic characteristics and PDGFRα expression

Baseline characteristics	No stain(n=33)	Positive(n=56)	Strong Positive(n=6)	*P*
**Age, years, median (range)**	54 (33-71)	53 (29-75)	47 (34-63)	*0.337*
**Gender (M:F)**	29:4	46:10	3:3	0.092*
**Etiology of Liver Disease**				*0.181*
HBV	25 (75.8)	45 (80.4)	3 (50.0)	
HCV	0 (0.0)	5 (8.9)	1 (16.7)	
Alcohol	2 (6.1)	1 (1.8)	0 (0.0)	
Others	6 (18.2)	5 (8.9)	2 (33.3)	
**Serum AFP, ng/mL (%)**				0.044*
≤200	19 (57.6)	38 (67.9)	1 (16.7)	
>200	15 (42.4)	18 (32.1)	5 (83.3)	
**Tumor number (%)**				*0.453*
Single	6 (18.2)	7 (12.5)	0 (0.0)	
Multiple	27 (81.8)	49 (87.5)	6 (100)	
**Tumor size**				*0.954*
≤5cm	22 (66.7)	39 (69.6)	4 (66.7)	
>5cm	11 (33.3)	17 (30.4)	2 (33.3)	
**Existence of satellite nodule**	1 (3.0)	9 (16.1)	0 (0.0)	*0.105*
**Existence of microvascular invasion (%)**	3 (9.1)	11 (19.6)	2 (33.3)	*0.235*
**Existence of LN metastasis (%)**	1 (3.0)	1 (1.8)	0 (0.0)	*0.863*
**Edmonson Grade (%)**				*0.284*
Grade 1, 2	18 (54.5)	27 (48.2)	1 (16.7)	
Grade 3, 4	15 (45.5)	29 (51.8)	5 (83.3)	
**Existence of Pathologic Cirrhosis (%)**	20 (60.6)	42 (75.0)	2 (33.3)	0.068*

PDGFRα, platelet-derived growth factor α; AFP, alpha-fetoprotein; LN, lymph node, Percent is calculated within the group with the similar PDGFRα expressing patients; *P values that are <0.1 are considered to be significant.

### Expression of PDGFRα in tumor and reciprocal non-tumor sites: tissue microarray study

PDGFRα expression was evaluated by a single pathologist, blinded to the patients' clinical information. The assessment was done in both tumor and matching non-tumor site of each patient (Table 3). There was a significant difference in PDGFRα expression of tumor and non-tumor sites and strong expression of PDGFRα was not seen in non- tumor sites.

**Table 3:** PDGFRα expression in paraffin sections

N=95	Tumor Site				Non-Tumor Site				P*
	No stainN (%)	WeakN (%)	ModerateN (%)	StrongN (%)	No stainN (%)	WeakN (%)	ModerateN (%)	StrongN (%)	
PDGFRα	19(20.0)	14 (14.7)	56(58.9)	6(6.3)	4(1.2)	19(20.0)	72(75.8)	0(0.0)	0.000*

PDGFRα, platelet-derived growth factor α; * *P* values that are <0.05 are considered to be significant when PDGFRα expressions are compared between tumor and non-tumor sites.

**Figure 1:**
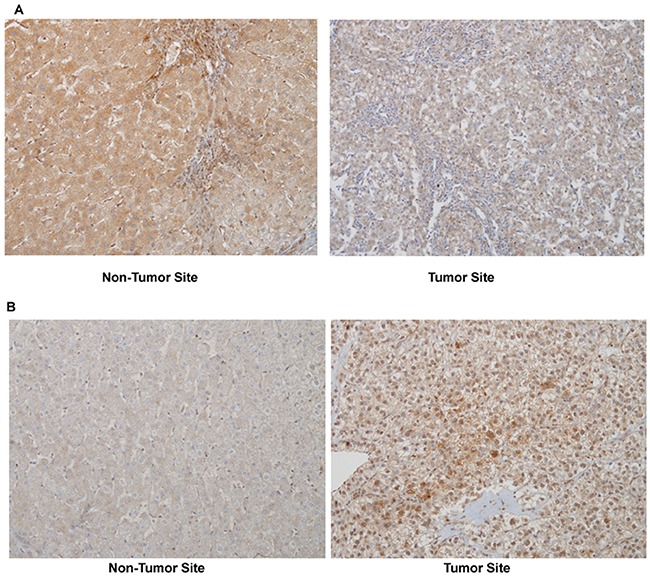
A representative PDGFRα expression in non-tumor and tumor site of the same patient (immunohistochemistry, original magnificationX200) **(A)** Moderate PDGFRα expression in non-tumor site with no stain in tumor site, and **(B)** no stain in non-tumor site with strong stain in tumor site.

PDGFRα expression is high in embryonic liver and then declines to minimal levels in adult hepatocytes [14]. On the other hands, PDGFRα expression is known to be immensely increased in cirrhotic liver, mainly on αSMA positive non-parenchymal cells [15]. We evaluated whether PDGFRα expression in tumor sites were associated with underlying liver cirrhosis or non-tumor site PDGFRα expression (Table 4). The cases with weak intensity of PDGFRα stain were classified as negative in this analysis. Among 95 patients, 62 patients (65.3%) showed positive for PDGFRα on tumor sites. PDGFRα positivity on tumor sites was not associated with existence of pathologically detected liver cirrhosis on matching non-tumor site. In addition, expression of PDGFRα on tumor sites had no relation with appearance of PDGFRα on reciprocal non-tumor sites (Table 4, Figure 1).

**Table 4:** Association of PDGFRα in tumor site and liver cirrhosis

N=95	Tumor Site		
	PDGFRα (−)	PDGFRα (+)	
Non-tumor site			
PDGFRα (−)	6 (6.3%)	17 (17.9%)	P=0.451
PDGFRα (+)	27 (28.4%)	45 (47.4%)	
Pathologic Liver Cirrhosis			
(−)	13 (13.7%)	18 (18.9%)	P=0.610
(+)	20 (21.1%)	44 (46.3%)	

Number of the patients from the total patients (n=95) is expressed in percent.

PDGFRα, platelet-derived growth factor α.

### Association of PDGFRα expression and the clinical outcome after the curative HCC resection: tissue microarray study

**Figure 2:**
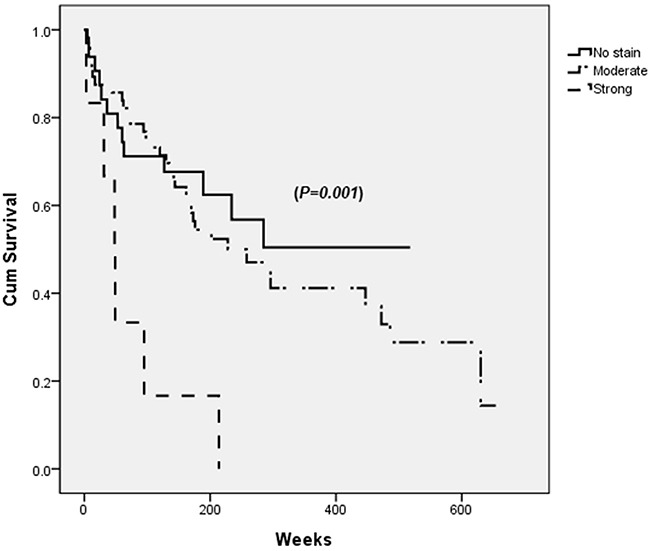
Survival after the curative resection of hepatocellular carcinoma (HCC) according to PDGFRα expression in tumor site Patients with strong PDGFRα expression in tumor site has lowest survival rate (dashed line) when analyzed by log-rank test (*p*=0.001).

Strong PDGFRα expression in tumor site was associated with decreased overall survival after curative HCC resection (*p=0.001*) (Figure 2).

Multivariate analysis test suggested that preoperatively elevated AFP above 200 ng/mL, existence of macrovascular invasion, having underlying liver diseases due to alcohol abuse were associated with poor survival in addition to strong PDGFRα positivity on tumor sites (Table 5).

**Table 5:** Analysis of factors associated with overall survival after hepatocellular carcinoma resection in chronic HBV patients

	Univariate	Multivariate	P*
	P*	Hazard Ratio (95% CI)	
**Gender, (Male Sex)**	*0.404*		
**Age, (<45 vs ≥45)**	*0.341*		
**AFP, ng/mL, (≥200)**	0.002*	**2.120 (1.143-3.930)**	0.017*
**Existence of liver cirrhosis**	*0.531*		
**Tumor size, (≤5cm vs >5cm)**	*0.139*		
**Tumor number, (single vs multiple)**	*0.304*		
**Existence of satellite nodule**	*0.643*		
**Edmonson grade, (>2)**	0.003*		
**Existence of microvascular invasion**	0.005*		
**Existence of macrovascular invasion**	0.003*	**6.016 (1.293-27.990)**	0.022*
**LN metastasis**	0.003*		
**Etiology of liver disease (Alcohol)**	0.039*	**8.945 (2.387-33.525)**	0.001*
**Expression of PDGFRα (Strong)**			
Tumor site	0.001*	**5.462 (1.799-16.582)**	0.003*
Non-tumor site	*0.171*		

AFP, alpha-fetoprotein; LN, lymph node; PDGFRα, platelet-derived growth factor receptor α, CI, confidence interval; *P values that are <0.05 are considered to be significant.

**Figure 3:**
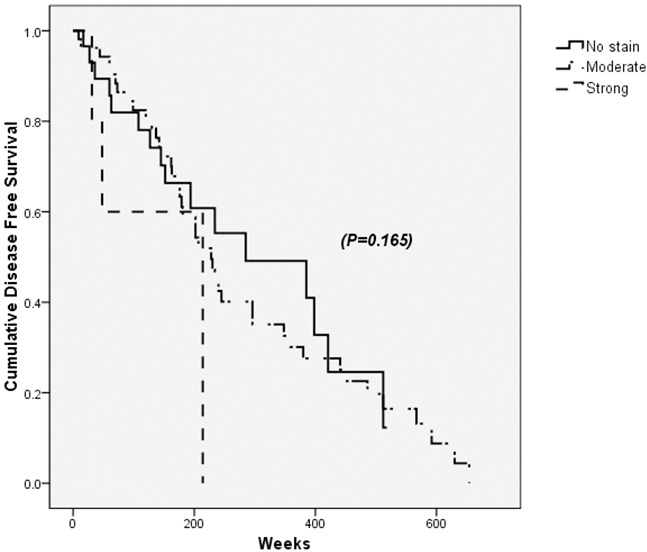
Disease free survival after the curative resection of hepatocellular carcinoma (HCC) according to PDGFRα expression in tumor sites PDGFRα positivity on tumor sites was not associated with HCC recurrence and disease free survival after curative resection (*p*=0.165).

PDGFRα positivity on tumor sites was not associated with HCC recurrence after curative resection (*p*=0.165) (Figure 3). Instead, multiple tumor mass, histologically high Edmonson grade and existence of macrovascular invasion were factors suggesting recurrence (Table 6).

**Table 6:** Analysis of factors associated with disease-free survival after hepatocellular carcinoma resection in chronic HBV patients

	Univariate	Multivariate	P*
	P*	Hazard Ratio (95% CI)	
**Gender, (Male Sex)**	*0.599*		
**Age, (<45 vs ≥45)**	*0.583*		
**AFP, ng/mL, (≥200)**	0.098*		
**Existence of liver cirrhosis**	*0.673*		
**Tumor size, (≤5cm vs >5cm)**	0.040*		
**Tumor number, (single vs multiple)**	0.011*	**3.378 (1.172-9.742)**	***0.024***
**Existence of satellite nodule**	*0.449*		
**Edmonson grade, (>2)**	0.002*	**2.324 (1.135-4.761)**	***0.021***
**Existence of microvascular invasion**	*0.283*		
**Existence of macrovascular invasion**	0.000*	**48.382 (6.303-371/364)**	***<0.000***
**Existence of LN metastasis**			
**Etiology of liver disease (Other than virus or alcohol)**	*0.546*		
**Expression of PDGFRα (Strong)**			
Tumor site	*0.165*		
Non-tumor site	*0.957*		

AFP, alpha-fetoprotein; LN, lymph node; PDGFRα, platelet-derived growth factor receptor α, CI, confidence interval; *P values that are <0.05 are considered to be significant.

### Association of tumor site PDGFRα and fibrosis or cancer-associated fibroblast related genes

In order to evaluate whether PDGFRα expression on tumor site has association with genes for liver fibrosis or cancer-associated fibroblast, freshly frozen HCC specimens with matching non-tumor sites were used for mRNA quantification. Gene expression on normal liver, obtained from non-tumor sites of resected liver due to colon cancer metastasized to the liver, served as the control. Summary of the patients with PDGFRα mRNA expression in tumor and non-tumor site is described in Table 7.

**Table 7:** Summary of hepatocellular carcinoma patients under fresh liver tissue evaluation

Patient No	Etiology of Liver Disease	AFP* (ng/dL)	Liver Cirrhosis†	PDGFRα RNA fold change‡	
				Non-tumor	Tumor
1	Others	9.9	YES	5.1836	2.0229
2	HBV	2.2	NO	1.1126	0.2511
3	HBV	4.0	NO	1.2189	2.3278
4	HBV	10.5	YES	8.3916	0.1800
5	HBV	159.4	YES	3.9832	0.6502
6	HBV	4.5	NO	1.3416	0.0338
7	HBV	3.9	NO	0.3515	0.8131
8	HBV	308.0	NO	2.2060	0.001
9	HBV	222336.0	NO	3.1909	2.5517
10	HBV	3.1	YES	1.9305	1.9744
11	Others	2.1	NO	0.0836	2.2214
12	HBV	24	YES	6.0166	0.1916
13	HBV	5.3	NO	1.8615	0.0161
14	HBV	3.3	YES	11.5230	2.1494
15	HBV	29.70	NO	4.8955	24.1081
16	HCV	57.5	YES	11.6434	40.6855

*Preoperative serum AFP.

† Liver cirrhosis is detected by pathologic evaluation.

‡ PDGFRα RNA fold changes in tumor and non-tumor sites are expressed in relative to PDGFRα expression in normal liver from specimens from patients with metastatic colon cancer.

Tumor site PDGFRα expression was correlated with increased *collagen α1(I) (Col1α(I))* mRNA on tumor site (Table 8). Increased PDGFRα mRNA was also associated with increased *lecithin retinol acyltransferase (Lrat)*, which is the marker for both activated and quiescent HSCs, and elevated *smooth muscle α-actin (αSMA)* expression, which is known as the marker for activated HSC and cancer-associated fibroblast. However, increased tumor site PDGFRα appeared to have no relation with non-tumor site *PDGFRα*, *Col1α(I)*, and *αSMA* expression.

**Table 8:** Correlation between PDGFRα in tumor sites and fibrosis or cancer-associated fibroblasts related genes

Genes	Correlation with PDGFRα in Tumor sites	*P* value
Tumor Sites		
*Col1α(I)*	0.472	***0.023***
*Lrat*	0.494	***0.017***
*αSMA*	0.497	***0.016***
*LOXL2*	0.142	0.517
Non-Tumor Sites		
*Col1α(I)*	0.233	0.284
*Lrat*	0.228	0.295
*αSMA*	0.160	0.466
*LOXL2*	0.221	0.310
*PDGFRα*	0.245	0.260

Col1α(I), collagen α1(I); Lrat, lecithin retinol acyltransferase; αSMA, α smooth muscle actin; LOXL2, lysyl oxidase homolog 2.

### Association of underlying liver cirrhosis and gene expression pattern

**Figure 4:**
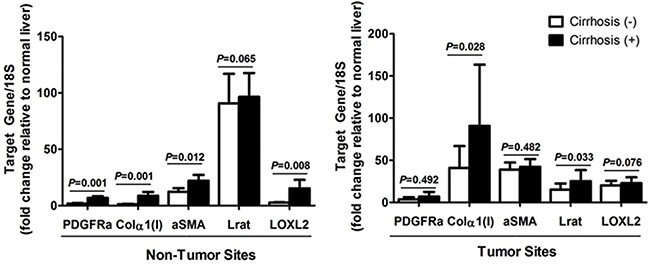
Expression of PDGFRα and other fibrosis related genes in (A) non-tumor sites, and (B) tumor sites according to the existence of liver cirrhosis Freshly frozen HCC specimens with matching non-tumor sites were used for mRNA quantification. Gene expression on normal liver, obtained from non-tumor sites of resected liver due to colon cancer metastasized to the liver, served as the control.

Since *PDGFRα*, *Col1α(I), Lrat, αSMA*, and *lysyl oxidase homolog 2 (LOXL2)* are known as the marker for liver fibrosis, expression of these genes in non-tumor site was assessed in association with the existence of liver cirrhosis [16]. The analysis showed that except Lrat, which is also a marker for quiescent HSC, expression of *Col1α(I), αSMA*, and *LOXL2* increased with accompanying liver cirrhosis in non-tumor site (Figure 4). On the other hands, expression of *PDGFRα* and *αSMA* in tumor site did not affected by underlying liver cirrhosis.

## DISCUSSION

In accordance with other previous studies [14, 17, 18], our study also demonstrated that strong PDGFRα expression in tumor sites was associated with poor survival outcome after HCC resection. However, the expression of PDGFRα did not necessarily associated with underlying liver fibrosis/cirrhosis or the expression of PDGFRα in adjacent non-tumor site of the liver. This result suggests that PDGFRα positivity in HCC does not necessarily associated with the process of liver fibrosis/cirrhosis in the background liver, but demonstrates the unique characteristics of HCC with poor prognosis.

PDGFRα is reported to be up-regulated in the injured liver with fibrosis and beginning to be recognized as a potential mediator of HSC activation, leading to liver fibrosis [15, 19, 20]. PDGFRα mRNA is highly expressed in αSMA positive HSCs, an important cellular component in the liver that contributes to generation and progression of fibrosis [21]. A recent study also demonstrated that reducing PDGFRα signaling in heterozygous PDGFRα mice showed alleviation of liver fibrosis from CCl_4_ injury [15]. On the other hands, excessive activation of PDGFRα signaling in PDGF-C transgenic mice resulted in liver cirrhosis by 9 months, and interestingly, these mice with cirrhosis develop liver cancer in the later stage, resembling human HCC with background liver cirrhosis [13]. In our study with human HCC samples, tumor site PDGFRα expression does not necessarily associated with the underlying liver cirrhosis and PDGFRα expression in the adjacent non-tumor sites.

Although PDGFRα expression in HCC may not be the consequence of liver fibrosis/cirrhosis, it should be emphasized that this suggestion does not preclude the possible communication between HSCs and tumor cells in the liver. HSC is one of the most important cellular components in the liver that contributes to generation and progression of fibrosis [22]. Upon liver injury, quiescent HSC transdifferentiate to extracellular matrix-producing myofibroblasts and overexpress various genes which is regarded as fibrosis related markers such as α-SMA and PDGFRα [23]. With the decades of the study, it has been widely recognized that HSCs seem to contribute in HCC progression [24, 25], when mechanism of this contribution is yet to be delineated.

In other solid tumors including pancreas cancer or intrahepatic cholangiocarcinoma, the abnormal activity of neoplastic epithelium is thought to stimulate stromal fibroblasts, resulting in the cancer associated desmoplastic response [26]. These abnormally stimulated fibroblasts are α-SMA positive cells and often designated as “cancer-associated fibroblasts (CAF)”. In cases of HCC, α-SMA positive HSCs contribute generation of liver fibrosis and cirrhosis in non-neoplastic liver, and previous HCC molecular profiling study demonstrated that the gene signature of the non-tumor tissue, adjacent to the tumor site, contains critical molecular information on HCC prognosis [3]. Although this signature study supports the important role of fibrosis/cirrhosis as a microenvironment generating HCC, this does not indicate that HSCs are the cancer-associated fibroblasts in HCC. It may not be even appropriate to conclude that PDGRα expressing HSC are the source of PDGFRα in HCC with poor prognosis.

The source of PDGFRα in HCC still carries many controversies and disputes. While several studies reported that PDGFRα positive cells in tumor sites are malignant cells themselves [14, 17, 18], another study argued that those were invaded non-parenchymal cells that showed PDGFRα positive [15]. In this study, we could not clearly differentiate PDGFRα positive non-parenchymal cells from hepatocytes since PDGFRα positive specimens demonstrated extensive brown staining in the target sections. However, when the same staining technique was applied, in PDGFRα negative sections, we could still identify some brown stained non-parenchymal cells with negatively stained parenchymal cells. In addition, a previous study demonstrated that overexpression of PDGFRα in Hep3B promoted cell proliferation, migration and invasion that are hallmarks of cancer cells [18]. Further studies on PDGFRα in HCC are expected to settle this dispute on the source of PDGFRα in HCC.

Although non-parenchymal cells in HCC might not be the major source of PDGFRα, non-parenchymal cells, especially HSCs, still seem to play an important role in PDGFRα positive HCC. Our study showed that PDGFRα expression in tumor site was associated with increased Lrat expression in tumor site. Since Lrat can only be expressed in HSCs and not in CAF, it can be speculated that PDGFRα expression in HCC might be associated with increased recruitment of HSCs in the tumor site. Moreover, previous fate tracing study revealed that HSCs are the dominant myofibroblasts in toxic, cholestatic and fatty liver injury [27], and it can be speculated that rise of HCC might have stimulated adjacent HSCs, incorporating them within the tumor matrix. However, this tumor stimulated HSC activation might not necessarily be related with underlying liver fibrosis/cirrhosis.

Although studies suggested that strong expression of PDGFRα was associated with decreased survival [14, 17, 18], the proportion of PDGFRα strong positive patients showed some discrepancies. This study and another previous study reported that PDGFRα consisted only about 6-7% [17], when the other studied described that high PDGFRα was seen in about 38% of HCC patients [18]. Very low proportion of PDGFRα strong positive patients would hinder the usefulness of PDGFRα as HCC target. On the other hands, it has been investigated that overexpression of vascular endothelial growth factor (VEGF) which was shown in about 20% of HCC patients tended to result in shortened overall survival [17], and inhibition and MET in MET-positive HCC which consist about 30-40% of HCC patients resulted in decreased tumor burden [28, 29]. Further studies that provide more consistent information on the proportion of PDGFRα positive HCC would put PDGFRα as a target for HCC treatment in the future.

There are several limitations in this study. Firstly, for the tissue microarray analysis, the results are limited by small number of PDGFRα strong positive HCC specimens. As another previous study [17] reported, our study also showed that about 70% of HCC had PDGFRα positive. On the other hands, very small number of patient had strong PDGFRα positivity which predisposed poor prognosis after the curative liver resection. This study cannot explain the pathophysiological difference in moderately positive and strong positive expression of PDGFRα since this study is an observational study using human specimens. Secondly, estimation from frozen human liver specimens may be limited by small sample size. In addition, even though we used liver specimens from the resected liver due to colon cancer with liver metastasis, these specimens may not be truly normal due to the metastasized cancer cells. Thirdly, as we have stated earlier, our study could not clearly identify the source of PDGFRα in HCC and differentiated PDGFRα positive non-parenchymal cells from parenchymal cells.

In spite of these limitations, this study also showed that PDGFRα expression is a poor prognostic marker for HCC after the curative surgical treatment, independent of underlying liver cirrhotic condition. Although PDGFRα is known to be abundantly expressed in activated HSCs, PDGFRα in tumor sites was not associated with underlying liver fibrosis/cirrhosis. Instead, PDGFRα expression in HCC was accompanied by enhanced Lrat expression, suggesting increased HSC residence in tumor sites.

## MATERIALS AND METHODS

### Liver specimens and patient information

This observational, retrospective study used paraffin-embedded HCC samples from surgical resection and freshly frozen liver tissue archived at the Tissue Bank. This study was approved by the institutional review board of Inha University Hospital (Incheon, Korea) and Yonsei University College of Medicine Gangnam Severance Hospital (Seoul, Korea).

The paraffin-embedded 95 HCC samples were from the patients who underwent liver resection between January 2000 and August 2010 at Inha University Hospital. Patients were 29-75 years in age (range, 53.2 ± 10.0, mean ± standard deviation (SD)), consisted of 78 males and 17 females. Mean follow-up time after surgery was 50.0 ± 39.3 months (range, 0-108). These patients received no preoperative treatment for HCC and had curative liver resection. These 95 patients were regularly followed up for HCC recurrence. AFP measurements were done every 3 months, and dynamic CT was regularly performed with the interval no longer than 6 months. All the patients were followed until the time of death or for at least 12 months. When a new lesion was detected by abdominal CT, evaluation for HCC recurrence was performed. When the liver CT showed compatible findings with HCC with accompanied AFP elevation, recurrence was diagnosed. However, if vascular pattern was not typical on liver CT, liver MRI and/or hepatic angiogram (HA) was performed. When the nodule showed atypical pattern on all the imaging studies, the nodule was followed up with the interval no longer than 3 months. In case of elevated AFP without evidence of newly appeared lesion on CT, magnetic resonance imaging (MRI) and/or HA was performed. Histopathologic analysis was performed on whole tissue section and the variables recorded for each case included tumor size, differentiation according to Edmondson-Steiner grade, presence of multiple tumors, microvascular and major vessel invasion, and background cirrhosis.

The freshly frozen liver tissue was archived at the Tissue Bank of Yonsei University College of Medicine, Gangnam Severance Hospital after the acquisition of patients' consent. From the bank, 16 HCC specimens with matching non-tumor site liver tissue were used for the analysis. Non-tumor site of the liver tissue resected from colon cancer patients due to liver metastasis (n=7) served as the normal control. Patient with HCC had liver resection between January 2012 and December 2014. Patients were 24-69 years in age (range, 57 ± 10.6), consisted of 9 males and 7 females. Mean follow-up time after surgery was 34 ± 21.6 months (range, 2.5-36.7). These patients received no preoperative treatment for HCC and had curative liver resection.

### Tissue microarray construction

Core tissue biopsies were taken from paraffin-embedded donor blocks and arranged in corresponding recipient tissue-array blocks using homemade recipient agarose-paraffin blocks as previously described [30]. At least 2 cores were sampled from the target lesions. When there were multiple nodules, the largest nodule served as the target lesion of the tumor site. Target lesions consisted of tumor sites and corresponding non-tumor sites for each patient.

### Immunohistochemistry (IHC) analysis

Immunohistochemical stain using mouse mono-clonal antibody against PDGFRa (SC-338, Santa Cruz, CA, 1:50 dilution) was performed as previously described on other studies [31]. The result was interpreted in a semi-quantitative manner. In cases of positive cells < 5% was considered as negative stain, and those > 5% was positive. In the positive cases, the scoring was performed according to the strength of the immunostaining as weak staining, moderate staining, and strong staining.

### RNA extraction and gene expression analysis by quantitative real-time polymerase chain reaction (PCR)

Total RNA was extracted from frozen whole using Trizol reagent (Invitrogen, Carlsbad, CA, USA) or Qiagen mini columns (Quiagen Inc. Valencia, CA, USA) according to the manufacturer's protocol. RNA samples were quantified by spectrophotometry. The RNA integrity was assessed using agarose gel electrophoresis and ethidium bromide staining. The RNA samples were then diluted in RNase-free water and stored at −70°C until use. Five micrograms of RNA were reverse-transcribed using RNA PCR kit version 1.2 (Takara Bio Inc, Japan) according to the manufacturer's recommendations. Oligonucleotide primers and TagMan probe for *PDGFRα*, *Col1α(I)*, *Lrat*, *αSMA*, and *LOXL2* were used with 18S as an internal control. The probes were obtained from Applied Biosystems (Perkin-Elmer/PE Applied Biosystems, Forster City, CA, USA), purchased as a ready-for-use form in Assays-on-Demand Gene Expression Products. The TaqMan probes was labeled at the 5′ end with the reporter dye FAM and minor groove binder (MGB) nonfluorescent quencher on the 3′end. The quantitative PCR was performed in triplicate for each sample on Step One Plus Real Time System (Applied Biosystems). Each 20-μL reaction contained 10 uL of TaqMan Fast Universal Master Mix (Applied Biosystems, Darmstadt, Germany), 1 uL of Gene Expression Mix and 2 uL of cDNA diluted in 7 μL RNase-free water. The thermal cycler conditions were 20 seconds at 95°C, and 40 cycles of 5 seconds at 95°C followed by 20 seconds at 60°. Fold change of mRNA in target genes relative to the endogenous 18S control were calculated as suggested on previous studies [32].

### Statistical analysis

Categorical variables were compared using two-sided χ^2^-test or Fisher's exact test when appropriate, and continuous variables were compared using the independent sample *t*-test or Kruskall-Wallis test. Factors predicting HCC recurrence and survival were analyzed using Cox proportional hazard model. Variables with *p*<0.05 from two-tailed test in univariable analysis were included in the multivariable model. These models were considered using conditional selection procedures. Hazard ratios (HRs) were presented with 95 percent confidence interval. *P*<0.05 from two-tailed test was considered significant for multivariate analysis. Statistical analyses were performed using SPSS software version 15.0 (SPSS, Chicago, IL).

## Abbreviations

HCC: hepatocellular carcinoma
PDGF: platelet derived growth factor
HSC: hepatic stellate cell
PDGFR: platelet-derived growth factor receptor
SD: standard deviation
HA: hepatic angiogram
MRI: magnetic resonance imaging
IHC: immunohistochemistry
PCR: polymerase chain reaction
Col1α(I): collagen 1α(I)
Lrat: lecithin retinol acyltransferase
αSMA: smooth muscle α-actin
LOXL2: lysyl oxidase homolog 2
MGB: minor groove binder
HR: hazard ratio
VEGF: vascular endothelial growth factor

## References

[1] Alter HJ, Seeff LB (2000). Recovery, persistence, and sequelae in hepatitis C virus infection: a perspective on long-term outcome. Semin Liver Dis.

[2] Caldwell SH, Crespo DM, Kang HS, Al-Osaimi AM (2004). Obesity and hepatocellular carcinoma. Gastroenterology.

[3] Hoshida Y, Villanueva A, Kobayashi M, Peix J, Chiang DY, Camargo A, Gupta S, Moore J, Wrobel MJ, Lerner J, Reich M, Chan JA, Glickman JN (2008). Gene expression in fixed tissues and outcome in hepatocellular carcinoma. N Engl J Med.

[4] Bataller R, Brenner DA (2005). Liver fibrosis. J Clin Invest.

[5] Hernandez-Gea V, Friedman SL (2011). Pathogenesis of liver fibrosis. Annu Rev Pathol.

[6] Bonner JC (2004). Regulation of PDGF and its receptors in fibrotic diseases. Cytokine Growth Factor Rev.

[7] Andrae J, Gallini R, Betsholtz C (2008). Role of platelet-derived growth factors in physiology and medicine. Genes Dev.

[8] Breitkopf K, Roeyen C, Sawitza I, Wickert L, Floege J, Gressner AM (2005). Expression patterns of PDGF-A, -B, -C and -D and the PDGF-receptors alpha and beta in activated rat hepatic stellate cells (HSC). Cytokine.

[9] Pinzani M (1995). Platelet-derived growth factor receptor expression in hepatic stellate cells: how too much of a good thing can be bad. Hepatology.

[10] Wong L, Yamasaki G, Johnson RJ, Friedman SL (1994). Induction of beta-platelet-derived growth factor receptor in rat hepatic lipocytes during cellular activation in vivo and in culture. J Clin Invest.

[11] Thieringer F, Maass T, Czochra P, Klopcic B, Conrad I, Friebe D, Schirmacher P, Lohse AW, Blessing M, Galle PR, Teufel A, Kanzler S (2008). Spontaneous hepatic fibrosis in transgenic mice overexpressing PDGF-A. Gene.

[12] Czochra P, Klopcic B, Meyer E, Herkel J, Garcia-Lazaro JF, Thieringer F, Schirmacher P, Biesterfeld S, Galle PR, Lohse AW, Kanzler S (2006). Liver fibrosis induced by hepatic overexpression of PDGF-B in transgenic mice. J Hepatol.

[13] Campbell JS, Hughes SD, Gilbertson DG, Palmer TE, Holdren MS, Haran AC, Odell MM, Bauer RL, Ren HP, Haugen HS, Yeh MM, Fausto N (2005). Platelet-derived growth factor C induces liver fibrosis, steatosis, and hepatocellular carcinoma. Proc Natl Acad Sci U S A.

[14] Stock P, Monga D, Tan X, Micsenyi A, Loizos N, Monga SP (2007). Platelet-derived growth factor receptor-alpha: a novel therapeutic target in human hepatocellular cancer. Mol Cancer Ther.

[15] Hayes BJ, Riehle KJ, Shimizu-Albergine M, Bauer RL, Hudkins KL, Johansson F, Yeh MM, Mahoney WM, Yeung RS, Campbell JS (2014). Activation of platelet-derived growth factor receptor alpha contributes to liver fibrosis. PLoS One.

[16] Staten NR, Welsh EA, Sidik K, McDonald SA, Dufield DR, Maqsodi B, Ma Y, McMaster GK, Mathews RW, Arch RH, Masferrer JL, Souberbielle BE (2012). Multiplex transcriptional analysis of paraffin-embedded liver needle biopsy from patients with liver fibrosis. Fibrogenesis Tissue Repair.

[17] Patel SH, Kneuertz PJ, Delgado M, Kooby DA, Staley CA, El-Rayes BF, Kauh JS, Sarmiento JM, Hanish S, Cohen C, Farris AB, Maithel SK (2011). Clinically relevant biomarkers to select patients for targeted inhibitor therapy after resection of hepatocellular carcinoma. Ann Surg Oncol.

[18] Wei T, Zhang LN, Lv Y, Ma XY, Zhi L, Liu C, Ma F, Zhang XF (2014). Overexpression of platelet-derived growth factor receptor alpha promotes tumor progression and indicates poor prognosis in hepatocellular carcinoma. Oncotarget.

[19] Borkham-Kamphorst E, Kovalenko E, van Roeyen CR, Gassler N, Bomble M, Ostendorf T, Floege J, Gressner AM, Weiskirchen R (2008). Platelet-derived growth factor isoform expression in carbon tetrachloride-induced chronic liver injury. Lab Invest.

[20] Martin IV, Borkham-Kamphorst E, Zok S, van Roeyen CR, Eriksson U, Boor P, Hittatiya K, Fischer HP, Wasmuth HE, Weiskirchen R, Eitner F, Floege J, Ostendorf T (2013). Platelet-derived growth factor (PDGF)-C neutralization reveals differential roles of PDGF receptors in liver and kidney fibrosis. Am J Pathol.

[21] Pinzani M, Milani S, Herbst H, DeFranco R, Grappone C, Gentilini A, Caligiuri A, Pellegrini G, Ngo DV, Romanelli RG, Gentilini P (1996). Expression of platelet-derived growth factor and its receptors in normal human liver and during active hepatic fibrogenesis. Am J Pathol.

[22] Li JT, Liao ZX, Ping J, Xu D, Wang H (2008). Molecular mechanism of hepatic stellate cell activation and antifibrotic therapeutic strategies. J Gastroenterol.

[23] De Minicis S, Seki E, Uchinami H, Kluwe J, Zhang Y, Brenner DA, Schwabe RF (2007). Gene expression profiles during hepatic stellate cell activation in culture and in vivo. Gastroenterology.

[24] Amann T, Bataille F, Spruss T, Muhlbauer M, Gabele E, Scholmerich J, Kiefer P, Bosserhoff AK, Hellerbrand C (2009). Activated hepatic stellate cells promote tumorigenicity of hepatocellular carcinoma. Cancer Sci.

[25] Zhao W, Zhang L, Yin Z, Su W, Ren G, Zhou C, You J, Fan J, Wang X (2011). Activated hepatic stellate cells promote hepatocellular carcinoma development in immunocompetent mice. Int J Cancer.

[26] Lee JI, Campbell JS (2014). Role of desmoplasia in cholangiocarcinoma and hepatocellular carcinoma. J Hepatol.

[27] Mederacke I, Hsu CC, Troeger JS, Huebener P, Mu X, Dapito DH, Pradere JP, Schwabe RF (2013). Fate tracing reveals hepatic stellate cells as dominant contributors to liver fibrosis independent of its aetiology. Nat Commun.

[28] Qi XS, Guo XZ, Han GH, Li HY, Chen J (2015). MET inhibitors for treatment of advanced hepatocellular carcinoma: A review. World J Gastroenterol.

[29] Koh YW, Park YS, Kang HJ, Shim JH, Yu E (2015). MET is a predictive factor for late recurrence but not for overall survival of early stage hepatocellular carcinoma. Tumour Biol.

[30] Kim KH, Choi SJ, Choi YI, Kim L, Park IS, Han JY, Kim JM, Chu YC (2013). In-house Manual Construction of High-Density and High-Quality Tissue Microarrays by Using Homemade Recipient Agarose-Paraffin Blocks. Korean J Pathol.

[31] Kang YK, Hong SW, Lee H, Kim WH (2010). Prognostic implications of ezrin expression in human hepatocellular carcinoma. Mol Carcinog.

[32] Livak KJ, Schmittgen TD (2001). Analysis of relative gene expression data using real-time quantitative PCR and the 2(T)(−Delta Delta C) method. Methods.

